# Vaginal Septoplasty in Septate Uterus with Double Cervix

**DOI:** 10.1155/2014/367360

**Published:** 2014-07-17

**Authors:** Samuel Barbanti, Nara Chiamulera, Beatriz Botelho

**Affiliations:** Gynecology and Obstetrics Service of Metropolitano Hospital, Medical School of Ingá Faculty (Uningá), Avenida D. Pedro I 65, 87110-001 Sarandi, PR, Brazil

## Abstract

Fusion defects of the Müllerian ducts occur frequently and they have been described by the American Fertility Society. However, septate uterus with cervical duplication and longitudinal vaginal septum is not described by this classification and has suggested a change in the classical theory of fusion of the Müllerian ducts. This paper describes a rare case report of a patient with complete septate uterus with double cervix and longitudinal vaginal septum, submitted to the vaginal septoplasty for dyspareunia, progressing to clinical improvement. The description of this case is to contribute with all uncommon cases of Müllerian anomalies reports and clinical treatment protocols, which is not yet established.

## 1. Introduction

The anatomical disorders of the female reproductive system occur frequently ranging from congenital absence of the vagina or uterus, until fusion defects of the Müllerian ducts. Generally, these changes are associated with genetic mutations, developmental disabilities, or environmental causes that have an effect on embryonic development stages [1].

The descriptions of Müllerian anomalies have changed over the years and they have been presently classified under those provided by the American Fertility Society. However, septate uterus with cervical duplication and longitudinal vaginal septum is not described by this classification [2]. Since McBean and Brumsted [3] described a similar case, the reports have suggested a change in the classical theory of unidirectional caudal to cephalic fusion of the Müllerian ducts.

In this context, this paper additionally brings a rare case report of a patient with complete septate uterus with double cervix and longitudinal vaginal septum, submitted to the vaginal septoplasty.

## 2. Case Presentation

**Figure 1:**
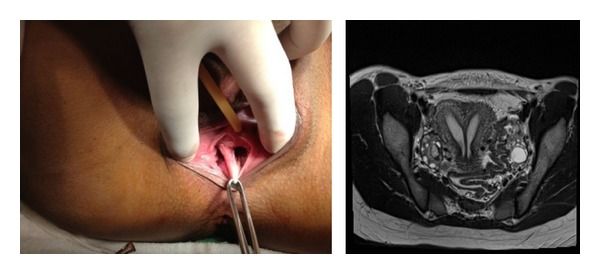
Complete septum evidenced two vaginal cavities (left). Pelvic image of magnetic resonance imaging showing septate uterus (right).

A 23-year-old primigravida presented to our service for missed abortion (8 weeks age). She reported menarche at 11 years old with normal pubertal development. Nowadays she complains dyspareunia associated with penetration difficulties. She did not report knowledge of urinary systems malformations and had no history of maternal exposure to diethylstilbestrol. Pelvic examination revealed normal external genitalia, with the presence of longitudinal vaginal septum and two uterine cervices (Figure 1). The magnetic resonance imaging (MRI), with intravenous contrast, showed the presence of uterus cavity with complete septate uterus and without indentations in uterine serous. Ultrasonography of the kidneys and urinary tract showed no changes. The patient underwent uterine evacuation.

Return to the service, the resection of vaginal septum was made with the patient in the dorsal lithotomy position and adequate vaginal retraction to allow exposure of the septum. Initially, the septum is grasped with Allis clamps, and a horizontal incision is made through the septum to resect. After 45 days of vaginal septoplasty, the patient reports improvement in dyspareunia.

## 3. Discussion 

Multiple anomalies involve the fusion of the Müllerian ducts, such as septate uterus, double cervix, and longitudinal vaginal septum. Traditionally the embryological events describe the fusion of the ducts occurring in the cranial-caudal direction, unidirectional [4]. However, the theory has been questioned by the reports of rare anomalies, such as presented in this paper [3, 5, 6]. In the case of septate uterus with cervical duplication and longitudinal vaginal septum, the anomaly suggests a failure in the fusion of the distal Müllerian ducts. In the presence of uterus and vaginal septum, the cervical duplication cannot be explained by the unidirectional theory. A new embryological theory, described initially by McBean and Brumsted [3], proposes that the fusion and resorption of ducts proceed in both directions, cranial and caudal. The embryological events are not clear yet and require further investigation. Brown and Badawy [7], in their case report, suggest that the system of classification of uterine anomalies, proposed by the American Society for Reproductive Medicine (ASRM), needs to be revised, because it does not include these cases.

The increasing number of described cases in the literature seems to be related to the availability of diagnosis. Several techniques have been used to investigate and confirm these cases, including hysteroscopy and laparoscopy, magnetic resonance imaging, and others, such as 3D ultrasound [5]. A precise diagnosis is necessary since the treatment and the reproductive outcome are dependent of anomaly. Patton et al. [8] published the largest series of cases of this anomaly, which reviewed diagnosis, clinical management, and reproductive outcome after surgical procedures. In symptomatic patients, as described in our case report, resection of the vaginal septum is important for well-being of dyspareunia penetration.

Ribeiro et al. [9] reviewed the cases of septate uterus, double cervix, and longitudinal vaginal septum, and the dysmenorrhea and infertility were the main symptoms in the majority of patients. Badalotti et al. [5] described a spontaneous pregnancy, with a healthy newborn at term. The description of the case suggests that surgical interventional treatments were observed only in women with a history of fetal loss, premature labor, and other obstetric complications which could be attributed to this anomaly. The prophylactic metroplasty of uterine septum in patients with no history of previous miscarriages or pregnancy complications is still under discussion and its impact on fertility was not elucidated yet.

Our expectative with the description of this case is to contribute with all uncommon cases of Müllerian anomalies reports. The septate uterus, double cervix, and longitudinal vaginal septum were not contemplated in the classification of the American Society for Reproductive Medicine and clinical treatment protocols have not been defined yet.
